# Flavonoid-Rich *Sambucus nigra* Berry Extract Enhances Nrf2/HO-1 Signaling Pathway Activation and Exerts Antiulcerative Effects In Vivo

**DOI:** 10.3390/ijms242015486

**Published:** 2023-10-23

**Authors:** Betul Cicek, Betul Danısman, Serkan Yildirim, Neslihan Yuce, Dragana Nikitovic, Ismail Bolat, Mehmet Kuzucu, Ertuğrul Ceyran, Ebru Bardas, Kirill S. Golokhvast, Aristidis Tsatsakis, Ali Taghizadehghalehjoughi

**Affiliations:** 1 Department of Physiology, Faculty of Medicine, Erzincan Binali Yildirim University, 24100 Erzincan, Turkey; betulll.cicek@gmail.com (B.C.); ebru.bardas@erzincan.edu.tr (E.B.); 2 Department of Biophysics, Faculty of Medicine, Ataturk University, 25240 Erzurum, Turkey; betul.danisman@atauni.edu.tr; 3 Department of Pathology, Faculty of Veterinary, Atatürk University, 25240 Erzurum, Turkey; syildirim@atauni.edu.tr (S.Y.); ismail.bolat@atauni.edu.tr (I.B.); 4 Department of Medical Biochemistry, Faculty of Medicine, Ataturk University, 25240 Erzurum, Turkey; neslihan.yuce@atauni.edu.tr; 5 Laboratory of Histology-Embryology, Faculty of Medicine, University of Crete, 71003 Heraklion, Greece; 6 Department of Biology, Faculty of Arts and Sciences, Erzincan Binali Yildirim University, 24100 Erzincan, Turkey; mkuzucu@erzincan.edu.tr; 7 Central Research and Application Laboratory, Agri Ibrahim Cecen University, 41000 Agri, Turkey; eceylan@agri.edu.tr; 8 Siberian Federal Scientific Centre of Agrobiotechnology RAS, 2B Centralnaya Street, 630501 Krasnoobsk, Russia; droopy@mail.ru; 9 Department of Forensic Sciences and Toxicology, Faculty of Medicine, University of Crete, 71003 Heraklion, Greece; tsatsaka@uoc.gr; 10 Department of Medical Pharmacology, Faculty of Medicine, Bilecik Şeyh Edebali University, 11230 Bilecik, Turkey; ali.tgzd@bilecik.edu.tr

**Keywords:** *Sambucus nigra* berries, indomethacin, gastric ulcer, Nrf-2/HO-1 signal pathway, IL-33

## Abstract

*Sambucus nigra* (SN) berry extract is characterized by high antioxidant and anti-inflammatory activity. The current study aimed to investigate the effect of SN berry extract against indomethacin (IND)-induced gastric ulcer in rats and the mechanism involved. SN berry extract alleviated IND-induced gastric ulcers, as shown by assessing pathological manifestations in the gastric mucosa. These protective effects are attributed to attenuated oxidative damage to the gastric mucosa, correlated to increased activity of superoxide dismutase (SOD), catalase (CAT), glutathione peroxidase (GPx), enhanced glutathione (GSH) levels, total antioxidant capacity (TAC), and upregulation of the Nrf2/HO-1 cascade. Moreover, oxidative stress markers, including malondialdehyde (MDA) and total oxidant status (TOS), were downregulated in SN-extract-treated animals. Furthermore, SN berry extract suppressed gastric mucosal inflammation by downregulating interleukin (IL)-33, IL-1β, IL-6, and tumor necrosis factor-alpha (TNF-α) levels, and attenuating myeloperoxidase (MPO) activity. The protective effects of SN berry extract were similar to those exerted by esomeprazole (ESO), an acid-secretion-suppressive drug. In conclusion, SN berry extract has antiulcerative effects, alleviating oxidative stress and inflammation.

## 1. Introduction

Non-steroidal anti-inflammatory drugs (NSAIDs) are widely prescribed medications worldwide. They generally treat pain, fever, and inflammation in various acute and chronic pathologies, including rheumatic disorders and osteoarthritis [1]. Unfortunately, the utilization of NSAIDs, such as indomethacin (IND), has been correlated to numerous gastrointestinal conditions, including the extent of gastric or duodenal ulceration, hemorrhage, and perforation, side effects that may eventually result in hospitalization or death [2]. Among the various types of NSAIDs, IND correlates with a higher risk of gastric ulceration [3]. 

Many studies have shown that IND exhibits prooxidant activity and promotes lipid peroxidation (LPO) by inducing the overproduction of reactive oxygen species (ROS) [4], thereby interfering with mucosal cells’ enzymatic and non-enzymatic antioxidant systems, including glutathione peroxidase (GPx), superoxide dismutase (SOD), and glutathione (GSH) [5].

Nuclear factor erythroid 2-related factor 2 (Nrf2) is the main target that modulates redox balance by stimulating the production of antioxidant enzymes [6]. Notably, heme oxygenase-1 (HO-1), one of the genes controlled by Nrf2, is closely associated with gastrointestinal tract protection [7]. Moreover, many antioxidant, anti-inflammatory, and immune-modulatory events are adjusted by HO-1 downstream products, suggesting that HO-1 induction could diminish proinflammatory cytokine signaling such as tumor necrosis factor-alpha (TNF-α) and interleukin-1β (IL-1β) [8,9].

IL-33 is a recently identified novel cytokine member of the IL-1 family whose biological effects are perpetrated via respective IL-1 receptor-like 1 (IL1RL1) binding [10]. Notably, IL1RL1 belongs to the key Toll-IL-1 receptor (TIR) superfamily, important in modulating the inflammatory response [11]. IL-33 is widely expressed, albeit in a cell-type-dependent manner [10]. Interestingly, in common with other epithelia with barrier-supporting functions, gastric epithelia highly express IL-33 [10]. 

Multifaceted roles have been attributed to IL-33 as, in addition to its function as a ligand cytokine, it has been characterized as a nuclear protein exerting transcriptional regulation [12,13]. Moreover, the effects of IL-33 have been correlated to the retainment of gastrointestinal system homeostasis. IL-33 is a cytokine noted for its role as a “danger” cue, e.g., an “alarmin”, and is released during tissue damage [14]. Although IL-33 is expressed in the stomach [15], its gastric function is not well established, and partially contrasting effects have been described. Thus, both in vitro and in vivo models of *Helicobacter pylori*-induced apoptosis showed protective effects of IL-33 [16]. Specifically, in gastric cells, IL-33 is suggested to increase cell growth, migration, and adhesion as well as to enhance their metabolic activity through caspase-3/Bcl-xL-dependent mechanisms [16]. Indeed, these authors indicate that IL-33 downregulates *H. pylori*’s harmful effects by attenuating the inflammatory response [16]. On the other hand, some studies suggest that the regenerative effects of IL-33 are attenuated in the presence of *H. Pylori* [17]. A separate study reports that IL-33 stimulates the release of proinflammatory cytokines such as IL-1β by gastric cells and TNF-α by mast cells in a gastritis mouse model. These findings correlate to gastric mucosa damage with a sustained inflammatory response, which delays the healing process in the ulcer area [18]. Furthermore, IL-33 promotes gastric epithelia spasmolytic polypeptide-expressing metaplasia (SPEM) [19,20]. IL-33 contributes to a repair response to mucosal injury but also to chronic damage and inflammation, leading to malignant transformation [21]. These studies, therefore, indicate that IL-33 is a crucial parameter in gastric mucosa homeostasis.

Proton pump inhibitors that suppress acid secretion, like esomeprazole (ESO), omeprazole, and ranitidine, have successfully prevented gastric mucosal damage caused by NSAIDs [22]. However, long-term use of these drugs has been putatively correlated to chronic side effects, including enhanced cardiovascular, liver, and kidney risk, disease, and neoplasms of the enteroendocrine system [23]. Therefore, developing reliable, non-toxic, and effective agents for gastric ulcer prevention and therapy is urgently required. 

Recently, researchers have increasingly focused on employing natural agents in preventing NSAID-induced gastric ulcers due to their antioxidant and anti-inflammatory features, with few or no side effects [24]. *Sambucus nigra* L. (SN) belongs to the Adoxaceae family and is used in folk medicine to treat respiratory infections and diabetes [25,26]. Thanks to these natural flavonoids, SN berries are characterized by high antioxidant and anti-inflammatory activity. Flavonoids are free radical scavengers that protect the human body against oxidative damage and inflammation [25,26]. The fruit of SN is an important source of phenolic compounds. Indeed, the primary polyphenols in SN berry extract have been identified as quercetin 3-*O*-(6″-acetyl-glucoside), kaempferol-3-*O*-glucoside, kaempferol-3-*O*-glucoside, kaempferol-3-*O*-rutinoside, and *p*-coumaric acid hexoside [27,28,29].

Considering the above-mentioned unique features, the ultimate target of this research was to explore the effects of SN berry extract on IND-related gastric ulcers in rats by comparing it with a reference drug (ESO). In addition, we focused on the identification of the possible mechanisms utilized by SN berry extract, with a particular emphasis on IL-33 and Nrf2/HO-1 signaling pathways.

## 2. Results

### 2.1. GC/MS Findings

**Figure 1:**
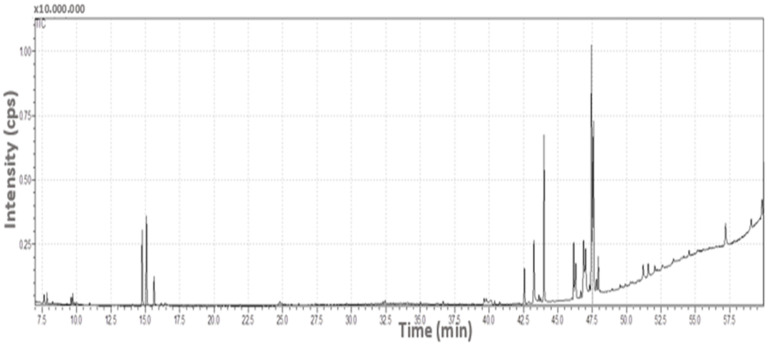
GC-MS chromatogram of SN berry extract separated with a Supelcowax 10 capillary column.

A GC-MS chromatogram of SN berry extract was obtained and is presented in Figure 1 and Table 1.

### 2.2. Quantitative Analysis of Phenolic and Flavonoid Compounds by LC-MS/MS

**Figure 2:**
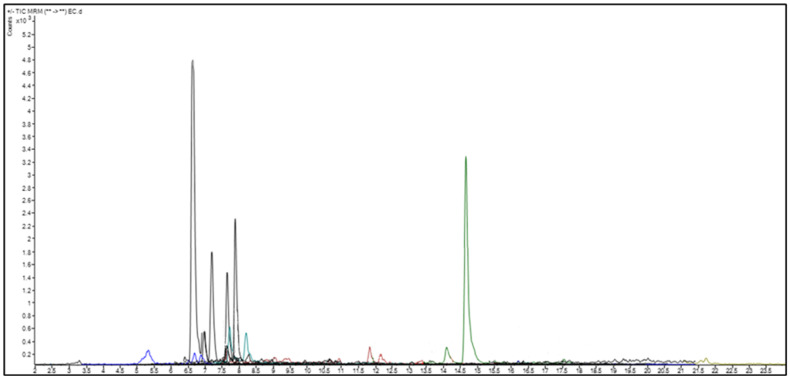
The *MRM* chromatogram of the analyzed SN berry extracts.

The analysis of phenolic and flavonoid content was carried out with Agillent brand 6460 Triple Quad model liquid chromatography–tandem mass spectroscopy (Figure 2). A standard chromatogram was obtained with pure standards (Supplementary Figure S1), and the amounts of analytes in the sample were determined by creating a calibration curve. The amounts of analytes are presented in Table 2. The gradient system used in the analysis is shown in Table S1.

In Table 3, the quantitative values of the compounds detected in the SN berry extract sample are presented.

### 2.3. Morphological Findings

**Figure 3:**
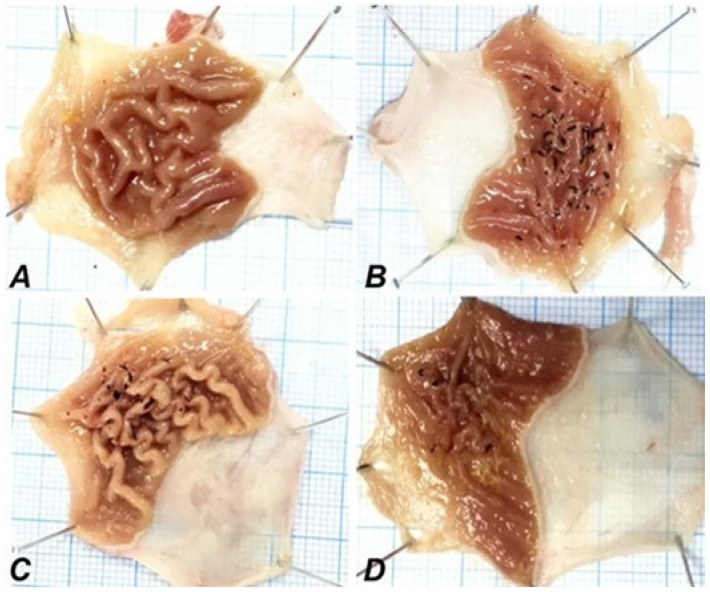
Rat gastric tissue, macroscopic appearance. (**A**) Control group demonstrating normal mucosa; (**B**) IND administration group with severely hemorrhagic ulcerated mucosal layer; (**C**) IND + ESO group; pre-treatment with ESO (20 mg/kg) effectively protected mucosal layer; (**D**) IND + SN group; pre-treatment with SN showing minor injuries and normal mucosa. IND: Indomethacin, ESO: Esomeprazole, SN: 350 mg/kg *Sambucus nigra* (SN) berry extract.

Rat stomach tissue was macroscopically examined, and the results are presented in Figure 3. As expected, no pathological findings were noticed in the control group. However, the administration of IND led to a severely thickened gastric wall and damaged epithelial lamina, ulceration, and hemorrhagic damage in the gastric mucosa. SN extract was administered at a dose of 350 mg/kg, selected in accordance with previous studies [30]. Additionally, the suitability of the respective dose was verified by morphological and histopathological analysis and the determination of ulcer index from preliminary pilot research performed in our lab. Pre-treatment with SN berry extract significantly reduced (*p* ˂ 0.05) erosion, ulceration, and hemorrhagic damage (Figure 3). The gastric ulcer index, score, and preventive index obtained from the macroscopic findings are presented in Table 4. Notably, SN berry extract exerted protective effects on the macroscopic gastric structure, e.g., erosion, ulceration, and hemorrhagic damage, similar to ESO (*p* ˂ 0.05).

### 2.4. IND-Induced Oxidative Stress Was Alleviated by Pre-Treatment with SN

**Figure 4:**
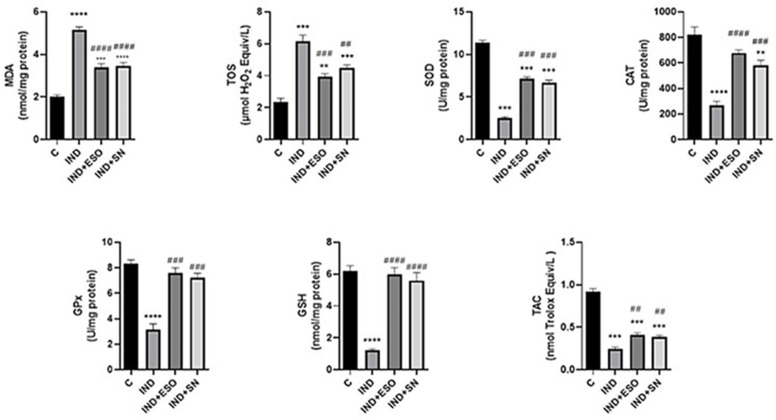
Effects of SN on oxidative stress markers in gastric tissues. Data are presented as means ± SD; ** *p* < 0.01, *** *p* < 0.001, **** *p* < 0.0001 versus control group; ## *p* < 0.01, ### *p* < 0.001, #### *p* < 0.0001 versus IND group. IND: Indomethacin, ESO: Esomeprazole, SN: 350 mg/kg *Sambucus nigra* (SN) berry extract, MDA: Malondialdehyde, TOS: Total oxidant status, SOD: Superoxide dismutase, CAT: Catalase, GPx: Glutathione peroxidase, GSH: Glutathione, TAC: Total antioxidant capacity.

As shown in Figure 4, administrating IND significantly (*p* < 0.0001) elevated MDA (5.140 ± 0.15; nmol/mg protein) levels compared with the normal control group (MDA: 2.025 ± 0.08 nmol/mg protein), well in accordance with previous studies [31]. In contrast, compared with rats in the IND group, rats pre-treated with ESO or SN exhibited strongly reduced MDA (3.38 ± 0.18 nmol/mg protein, 3.46 ± 0.15 nmol/mg protein, respectively) levels (*p* < 0.0001). In parallel to the MDA findings, the IND group demonstrated prominently upregulated (*p* < 0.001) TOS (6.16 ± 0.38 μmol H_2_O_2_ Equiv/L) levels in comparison to the control (2.34 ± 0.25 μmol H_2_O_2_ Equiv/L). However, rats pre-treated with ESO or SN berry extract exhibited significantly decreased TOS (3.92 ± 0.20 μmol H_2_O_2_ Equiv/L; 4.48 ± 0.19 μmol H_2_O_2_ Equiv/L, respectively) levels. 

In parallel with these findings, a reduction was detected in the activities of first-line defense antioxidant enzymes, e.g., SOD (from 11.42 ± 0.22 U/mg protein to 2.56 ± 0.12 U/mg protein; *p* < 0.001), CAT (from 821.0 ± 60.67 U/mg protein to 269.0 ± 31.60 U/mg protein; *p* < 0.001), and GPx (from 8.32 ± 0.3 U/mg protein to 3.13 ± 0.48 U/mg protein; *p* < 0.001). Likewise, the levels of the second-line defense antioxidant GSH (6.21 ± 0.33 nmol/mg protein) were strongly reduced in IND-treated ulcerated rats 1.23 ± 0.8 nmol/mg protein;); *p* < 0.0001. These depleted levels were replenished by pre-treatment with ESO or SN, which increased SOD, CAT, GPx, and GSH levels, compared with IND-induced ulcerated rats. SN berry-treated ulcerated rats demonstrated increased SOD (6.7 ± 0.26 U/mg protein; *p* < 0.001), CAT (583.2 ± 37.75 U/mg protein; *p* < 0.001), GPx (7.2 ± 0.36 U/mg protein; *p* < 0.001), and GSH (5.6 ± 0.5 nmol/mg protein; *p* < 0.001) levels when compared to the group treated just with IND. Also, a significant reduction in TAC levels was determined in the IND (0.25 ± 0.02 nmol Trolox Equiv/L; *p* < 0.001) group compared with the control group (0.92 ± 0.03 nmol Trolox Equiv/L), which was markedly reversed by SN berry extract administration (0.38 ± 0.02 nmol Trolox Equiv/L; *p* < 0.01). These effects were similar to those in rats treated with the control drug ESO.

### 2.5. IND-Induced Neutrophil Infiltration Was Improved by Pre-Treatment with SN Berry Extract

**Figure 5:**
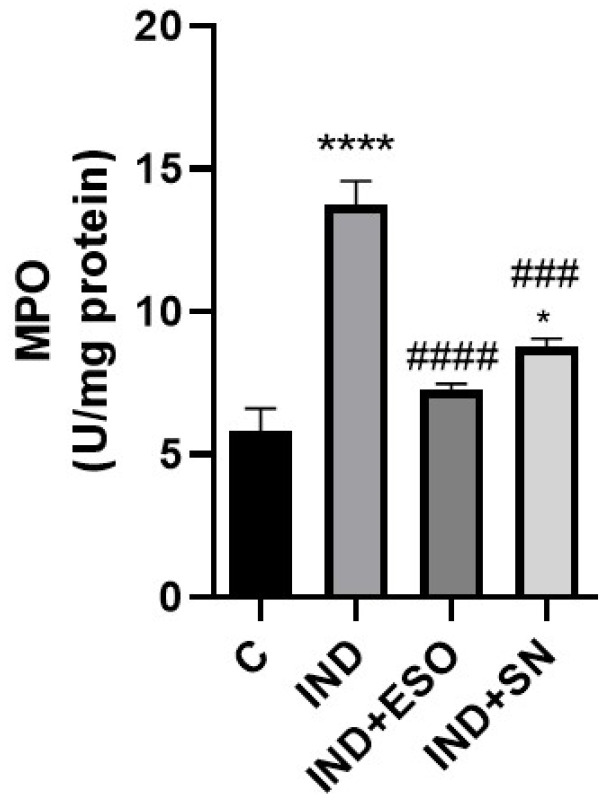
Effects of SN on gastric tissue MPO levels in control and experimental groups. Data are presented as means ± SD; * *p* < 0.05 **** *p* < 0.0001 vs. control group; ### *p* < 0.001, #### *p* < 0.0001 versus IND group. IND: Indomethacin, ESO: Esomeprazole, SN: 350 mg/kg *Sambucus nigra* (SN) berry extract, MPO: Myeloperoxidase.

The level of MPO, correlated with the influx of neutrophils, was markedly elevated in gastric tissue in response to IND administration as compared with control rats (13.72 ± 1.92; 5.82 ± 0.79 U/mg protein; *p* < 0.0001; respectively). MPO release was strongly attenuated by pre-treatment with SN berry extract (8.77 ± 0.3 U/mg protein; *p* < 0.001), as demonstrated by the significant decrease in gastric tissue MPO levels compared with IND-treated rats (Figure 5). These data suggest that SN extract inhibits neutrophile influx in SN-treated ulcerated rats. Meanwhile, ESO nearly normalized MPO levels (7.3 ± 0.22 U/mg protein; *p* < 0.001) (Figure 5).

### 2.6. The Effects of SN Berry Extract on Inflammation Factors 

**Figure 6:**
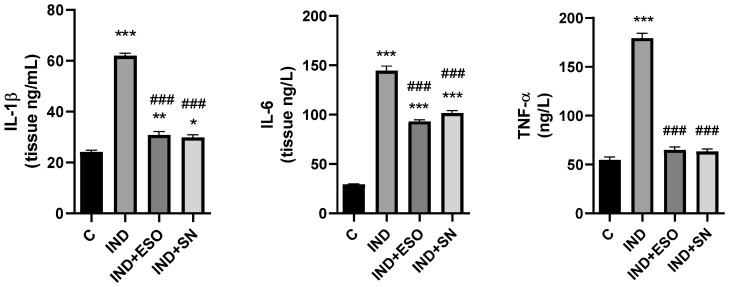
Effects of SN on inflammatory markers in gastric tissues. Data are presented as means ± SD; * *p* < 0.05, ** *p* < 0.01, *** *p* < 0.001 versus control group; ### *p* < 0001 versus IND group. IND: Indomethacin, ESO: Esomeprazole, SN: 350 mg/kg *Sambucus nigra* (SN) berry extract. IL-1β: Interleukin 1 beta, IL-6: Interleukin 6, TNF-α: Tumor necrosis factor alpha.

As shown in Figure 6, treatment with IND resulted in a prominent elevation in all inflammatory markers, including IL-1β (61.98 ± 0.98 ng/mL), IL-6 (144.6 ± 4.68 ng/L), and TNF-α (179.4 ± 4.97 ng/L) as compared to the control rats (24.11 ± 0.75 ng/mL; 29.25 ± 0.48 ng/L; 54.82 ± 2.88 ng/L, respectively) (*p* < 0.001). Meanwhile, pre-treatment with either SN berry extract or ESO attenuated the increased cytokine release (e.g., IL-1β (29.82 ± 1.15 ng/mL; 30.85 ± 1.4 ng/mL, respectively), IL-6 (101.6 ± 2.71 ng/L; 93.05 ± 1.7 ng/L, respectively) and TNF-α (63.23 ± 2.65 ng/L; 64.91 ± 3.02 ng/L, respectively) (*p* < 0.001) as compared to IND treated group. Likewise, SN berry extract exerted protective effects similar to the control drug (ESO). 

### 2.7. Effect of SN Berry Extract on Gastric Tissue Histopathology 

**Figure 7:**
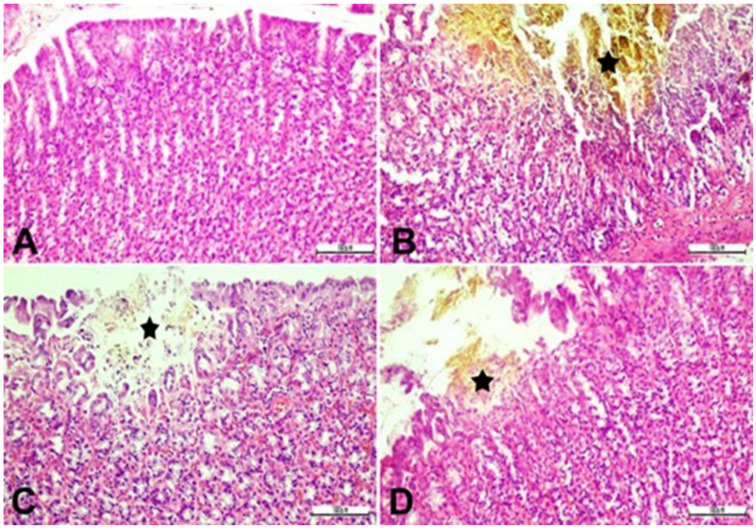
Histological images of gastric mucosa stained with H and E (×100). Stomach tissue, erosive and ulcerative areas (star) in the gastric mucosa epithelium, and inflammatory cell reactions in the mucosal layer. (**A**): Control group; (**B**) IND group; (**C**) IND + ESO group; (**D**) IND + SN group. IND: Indomethacin, ESO: Esomeprazole, SN: 350 mg/kg *Sambucus nigra* (SN) berry extract.

The effect of SN berry extract on the histology of the gastric tissue is shown in Figure 7. Histopathological examination of the control group gastric tissue revealed that tissue architecture was normal, with intact stomach glands. The mucosa contained normal surface epithelium and enclosed branched and tubular glands. As opposed to this, IND administration led to tissue damage, including severe submucosal edema, prominent desquamation of the epithelium, marked erosions and ulcerations in the gastric mucosa, widespread necrosis of the mucosal epithelium, and strong mononuclear cell infiltration in the necrotic regions in combination with abundant erythrocytes in the lumen. Furthermore, inflammatory cellular infiltration and severe hyperemia in the lamina propria were detected in the IND-treated rats. Pre-treatment with SN berry extract significantly attenuated the incidence of pathological lesions detected in the IND group (*p* ˂ 0.05). Thus, minor erosion, hyperemia, and focal necrosis of the stomach mucosa were determined. Like the SN group, ESO exhibited protection against the degeneration of gastric tissue (*p* ˂ 0.05) (Figure 7, Table 4).

### 2.8. Correlation of Histopathological Changes and IL-33 Expression-Effect of SN

**Figure 8:**
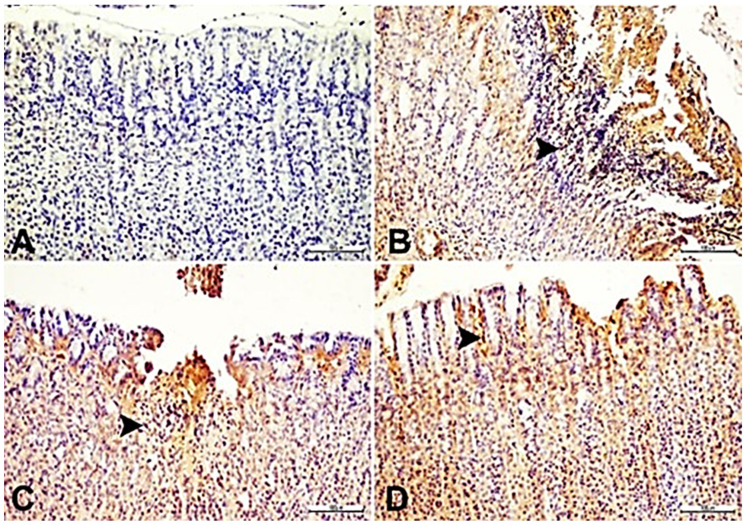
Representative photomicrographs showing IL-33 immunohistochemical staining (magnification 100×): stomach tissue; IL-33 expressions (arrowheads) in inflammatory cells. (**A**): Control group; (**B**) IND group; (**C**) IND + ESO group; (**D**) IND + SN group. IND: Indomethacin, ESO: Esomeprazole, SN: 350 mg/kg *Sambucus nigra* (SN) berry extract.

An examination of control sections demonstrated immunoreactivity levels to IL-33 in the gastric mucosal cells. The administration of IND induced an intense and marked positive immune reaction to IL-33, especially in the vicinity of necrotic regions, the cytoplasm of inflammatory cells, and surrounding vessels compared to the control (*p* ˂ 0.05). The SN and ESO groups exhibited an evident decline in IL-33 immunoreactivity around necrotic areas in the cytoplasm of inflammatory cells and surrounding vessels compared with the IND group (*p* ˂ 0.05) (Figure 8, Table 5).

### 2.9. Effect of SB Berry Extract Pre-Treatment on Nrf-2 and HO-1 Expression as Analyzed by Immunofluorescence 

**Figure 9:**
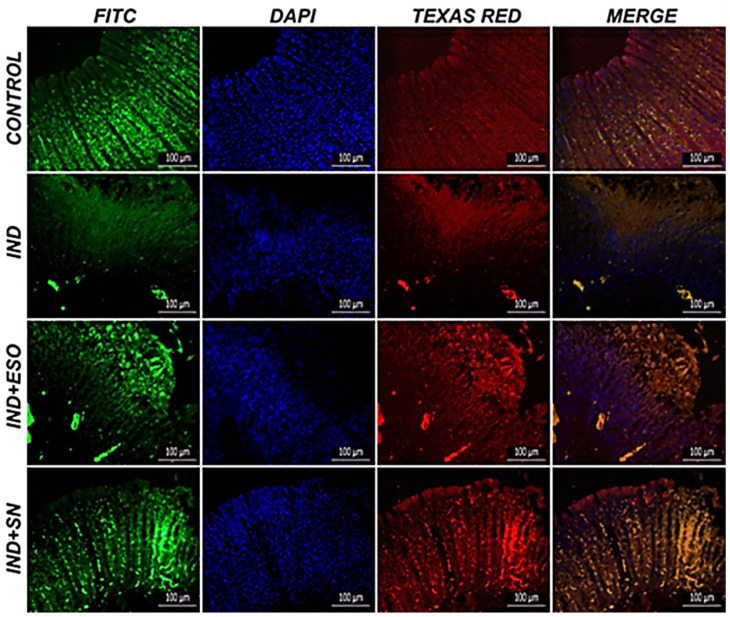
Double immunofluorescence labeling and merged images. Gastric tissue Nrf2 expression (FITC), HO-1 expression (Texas Red), D-IF, Bar: 100 µm. Stomach tissue Nrf2 (FITC) and HO-1 (Texas Red) expression in gastric mucosa cells. DAPI: 4′,6-diamidino-2-phenylindole, FITC: fluorescein isothiocyanate, IND: indomethacin, ESO: esomeprazole, SN: 350 mg/kg *Sambucus nigra* (SN) berry extract.

The control group exhibited normal Nrf2 and HO-1 expression. Severe gastric oxidative damage was induced by IND administration, as evidenced by the decrease in and aberrant deposition of Nrf2 and HO-1 immunoreactivity compared to the control group (Figure 9; *p* ˂ 0.05). On the other hand, pre-treatment with SN berry extract or ESO increased Nrf2 and HO-1 stain and normalized their distribution in gastric mucosa compared to the IND group (*p* ˂ 0.05). Table 6 presents the immunofluorescent findings.

## 3. Discussion

Treatment with NSAIDs results in disorders of the gastrointestinal tract in approximately 40% of the treated population. Moreover, in 13% of patients, NSAID administration is associated with the incidence of gastric ulcers. Thus, NSAID administration can cause adverse impacts on the patient [32]. Therefore, developing efficient NSAIDs without side effects remains an unmet medical need. Natural substances have been highlighted as a promising source for this line of research [33]. 

SN berry extract has exhibited pharmacological properties in several studies at doses from 15 mg/kg to 600 mg/kg. Its pharmacological effects can be diverse, depending on the ingredient concentration. Based on previous studies [30] and data obtained from our preliminary pilot research, 350 mg/kg SN berry extract was utilized in the present study. In addition, SN acts as an antioxidant, anti-inflammatory drug, and immunomodulator [25,26]. To the best of our knowledge, this is the first study to examine the effect of SN berry extract on gastric mucosa in IND-induced gastric ulceration rat models. Glycerol monostearate, catechin, kaempferol-3-O-glucoside, stearic, oxirane, vaccenic, and linoleic acid are some of the effective components in the SN berry extract for gastric and medical usage, as demonstrated in previous studies [34]. Moreover, earlier studies have shown the high antioxidant activity of SN berry extract, exhibiting an inhibition of 82.08 to 89.25% in relation to DPPH radicals. The antioxidant properties of SN berry extract have been verified to primarily depend on the presence of phenolic compounds [35].

IND can cause gastric injury via different mechanisms, including a reduction in bicarbonate release, which damages mucosal cell membranes, inducing cytotoxicity [36,37]. These cytotoxic effects cause the recruitment of leukocytes that release free radicals and PICs, subsequently, among others, reducing gastric blood flow and ultimately driving adenic cells to apoptosis [36]. This study observed multiple macroscopic hemorrhagic lesions and an elevated ulcer index in the IND group, consistent with previous studies [32,36,38,39]. Furthermore, vasodilation and occlusion of blood vessels lead to redness in hemorrhagic lesions, whereas HCl induced a brown discoloration. However, pre-treatment with SN berry extract retained normal stomach macroscopic structure and histology, showing that it exerts gastroprotective effects.

Likewise, studies have established that NSAIDs can cause gastric ulcers due to increased oxygen-derived free radical production and correlated oxidative stress. Subsequently, the radical peak overwhelms the antioxidant enzymes’ capacity, resulting in a decline in their activity [32]. In addition, free radicals interact with cell membranes to form lipid peroxides such as MDA [5]. This study verified the emergence of oxidative stress due to a disturbed redox balance in the IND-induced rat gastric ulcer model. Oxidative stress was identified as a reduction in SOD, CAT, GPx, GSH, and TAS levels correlated to a marked upregulation of MDA and TOS levels. Our findings relate well to previous studies, such as that by Kucukler et al., who showed that IND induces gastric injury by causing excessive ROS production and high MDA levels accompanied by an impairment of antioxidative enzyme activity [40]. 

Pre-treatment of the animals with SN berry extract or ESO reduced MDA and TOS levels and strengthened the antioxidant defense mechanism by elevating SOD, CAT, GPx, GSH, and TAS levels. Our findings, therefore, indicate that SN berry extract ameliorates gastric ulcers through an antioxidant mechanism associated with the scavenging of free radicals. Some previous studies have suggested that SN extract exerts protective effects by enhancing antioxidant activity in experimental diabetes mellitus [41]. 

In continuation, we focused on identifying the possible mechanisms of actions of SN berry extract. For this purpose, we focused on Nrf2/HO-1 signaling, a primary antioxidant defense system for oxidative stress. Indeed, the Nrf2/HO-1 signaling alteration observed in IND-induced gastric damage has been strongly associated with elevated ROS, increased proinflammatory cytokines, and an impairment of the epithelial integrity of the digestive mucosa [9,41]. The current study demonstrated that IND reduced Nrf2 expression concomitantly with downregulating the target protein HO-1, which is well in accordance with the impaired ability of the cell to respond to oxidative stress. Our findings were consistent with the study by Balaha et al., where these authors showed that IND represses Nrf2/HO-1 cascade concomitantly with decreased GSH level and SOD activity and elevated lipid peroxidation in rat gastric tissue [42], whereas pre-treatment with SN berry extract or ESO upregulated the levels of antioxidant enzymes, including SOD, CAT, GPx, and GSH, in harmony with upregulated activation of the Nrf2/HO-1 cascade. The holistic result of this mechanism is the enhanced ability of cells to resist gastric oxidative damage. Likewise, Lin et al. demonstrated that SN berry extract increases Nrf2/HO-1 signaling to enhance oxidative defense features in UVB-induced photoaging in human skin keratinocytes [43]. 

Crosstalk between oxidative stress and inflammation is crucial in the pathogenesis of IND-induced gastric injury [40]. Previous studies have demonstrated that IND can lead to inflammation by triggering polymorpho-nuclear neutrophils and macrophages to infiltrate the gastric mucosa, resulting in an enhanced release of proinflammatory cytokines and other mediators supporting further oxidative damage [42,44]. IND administration in the current study triggered a severe inflammatory response in the gastric mucosa, with an elevated generation of MPO consistent with previous experimental studies [41,45]. We also demonstrated that IL-33 expression is triggered by IND administration and that elevated IL-33 promotes elevated proinflammatory cytokine levels like TNF-α, IL-1β, and IL-6 in gastric tissue. Even though the relation of IL-33 to gastric ulcers is not well defined, Kuo et al. reported that IL-33 and IL-8 expression levels were markedly elevated in *H. pylori*-infected gastric epithelial cells [18]. A separate study of a rat model of gastric ulcers reported that IND prompted an extensive inflammatory response with upregulated levels of both IL-6 and TNF-α [9]. The present study revealed that SN berry extract and ESO suppressed the mucosal inflammatory response, decreasing gastric mucosal inflammatory cytokines, including TNF-α, IL-1β, IL-6, and MPO. These results coincide with SN berry extract’s anti-inflammatory properties previously shown in several experimental studies. SN ameliorated mice carrageenan-induced inflammation by suppressing inflammatory cytokine production, IL-1β, IL-6, and TNF-α [44]. In addition, Farrell et al. reported that SN berry extract reduced metabolic dysfunction in diet-induced obese mice through its anti-inflammatory activities by suppressing the generation of TNF-α and IL-6 [46].

These findings were confirmed by the histopathological analysis of IND-induced rats, which showed severe damage in the mucosal layer, severe erythrocytes in the lumen, gastric erosion, and ulceration, presence of inflammatory cells, and reduced epithelium integrity in gastric tissues, which is in accordance with previous reports [38,40]. Pre-treatment with SN berry extract or ESO prevented the incidence of severe damage to the stomach and recovered epithelial, mucosal, and submucosal permeability/integrity, as confirmed by our biochemical findings. Notably, Santin et al. previously showed an anti-inflammatory effect of SN berry extract by modulating macrophage and neutrophil functions, which supports our findings [45].

In conclusion, pre-treatment with SN berry extract attenuated IND-induced gastric damage in the rat model in a manner similar to ESO, the control drug. 

## 4. Materials and Methods

### 4.1. Chemicals and Reagents

IND (Endol 25 mg; 25 cap.) was purchased from DEVA Holding A.S. (Istanbul, Turkey); esomeprazole (ESO; Nexium 40 mg; 28 tablets) was procured from Astra-Zeneca Pharmaceutical Company (Istanbul, Turkey). Sephadex LH20 column was purchased from Merck (Darmstadt, Germany). IL-1 β, IL-6, and TNF-α were purchased from Elabscience (Houston, TX, USA). Total antioxidant capacity (TAC) and total oxidant capacity (TOC) colorimetric kits were purchased from Rel Assay Diagnostics (Gaziantep, Turkey). Hematoxylin–eosin (H and E) was obtained from Merck (Darmstadt, Germany). Nrf2 (Anti-Nrf2 antibody, Catalog #: ab31163), HO-1 (Anti-HO-1 antibody, Catalog #: ab13243), and IL-33 (Anti-IL-33 antibody, Catalog #: ab118503) were obtained from Abcam (Boston, MA, USA). Fluorescein-5-Isothiocyanate (FITC; secondary antibody, Catalog #: ab6785) and Texas Red (secondary antibody, Catalog #: ab6719) were purchased from Abcam (Boston, MA, USA).

### 4.2. Preparation of SN Berry Extract

*Sambucus nigra* L. berries were collected in Erzincan, Turkey (39.601729, 39.714934), between the dates 5 August 2021 and 10 August 2021. The harvested SN berries were promptly treated with solid carbon dioxide in isolation containers after being harvested and rinsed three times with 0.9 percent NaCl. Then, 20 g of lyophilized berries was taken, ground in a laboratory mill, and passed through a 0.5 mm sieve. In the extraction procedure, 5 g of the sample was subjected to extraction using 100 mL of ethanol:water (4:1) mixture. This solvent mixture was acidified with 0.1% *v*/*v* formic acid prior to extraction. The extraction process was carried out in a shaking incubator set at 3 rcf, maintained at 4 °C, and continued for 24 h in the dark. The extract was concentrated in a rotary evaporator after passing through a filter with a pore diameter of 0.22 µm. The resulting extract was subjected to Sephadex^®^ LH-20 column chromatography with methanol as the mobile phase and flavonoid/phenolic-containing eluates were collected. The mixed eluates were then concentrated in a rotary evaporator. The concentrated extract was stored for qualitative screening by means of MS/MS and LC-MS/MS systems.

### 4.3. GC/MS and LC-MS/MS Analysis

After preparing an appropriate dilution of SN berry extract with methanol, it was filtered with a 0.22 µm PTFE filter and transferred to a vial. The volatile component screening in the extract was performed with a Shimadzu QP2010 Ultra Gas Chromatography–Mass Spectroscopy. A 60 m × 0.25 mm × 0.25 μm (Teknokroma-TBR 5MS) film column was used as the column. Flow was 1.1 mL/min, injection volume was 1 μL, and injection temperature was set at 250 °C. Electron Impact ionization was used as the ionizator, and the scanning range of the mass analyzer was set as 40–600 *m*/*z*. The temperature program applied in the analysis was as shown in Table 7. The mass spectra of each peak in the obtained chromatogram (in the range of 50–650 *m*/*z*) were matched with the library data. 

The analysis of phenolic and flavonoid content was carried out with Agilent brand 6460 Triple Quad model liquid chromatography–tandem mass spectroscopy. The C18 column (250 mm × 4.6 mm, 5 μm) was used for chromatographic separation. The flow rate was 0.4 mL/min, and the injection volume was 10 µL. The column temperature was set to 30 °C. Formic acid solution at a concentration of 0.1% (*v*/*v*) (A) and methanol (B) were used as the mobile phase. The gradient system used in the analysis is shown in Table S1. A standard chromatogram was obtained with pure standards (Supplementary Figure S1), and the amounts of analytes in the sample (Table 1) were determined by creating a calibration curve. 

### 4.4. Gastric Ulcer Model

The gastric ulcer model was developed as previously reported in the literature [42]. Briefly, rats were not permitted to eat but were granted access to water, which was also removed one hour before the stimulation of the gastric ulcer to ensure that the rats’ stomachs were empty. Following this, the rats were given a single dose of 100 mg/kg of IND to induce gastric ulcers. Various degrees of ulceration were determined 4 h after administration of IND. 

### 4.5. Experimental Design

A total of 28 Wistar albino male (250–300 g) rats were initially maintained under normal conditions (24 ± 1 °C with a 12:12 h reverse lighting cycle) with free attainment to chow and water. The rats were randomly separated into four groups (n = 7). Group I (Control) and Group II (100 mg/kg IND) were given a vehicle (saline) for 14 days. Group III (IND + ESO) was pre-treated orally with 20 mg/kg/day of ESO for 14 days, the standard treatment [23]. Group IV (IND + SN) was treated orally with 350 mg/kg SN berry extract (most effective dose) in saline for 14 days. Gastric ulceration was induced on day 14 of the experiment by 100 mg/kg IND in rats in Groups II–IV. SN extract was administered at a dose of 350 mg/kg, selected in accordance with previous studies [30]. The suitability of the dose was verified by the findings of morphological and histopathological analyses and the determination of ulcer index was obtained by preliminary pilot research performed in our lab. 

### 4.6. Morphological Examination 

Macroscopic evaluation of gastric tissue samples was performed. The mucous layers were examined and macroscopic images of the stomach tissues were taken. GU index was evaluated by making measurements in TrueView, Olympus program. The preventive index was calculated as the Ulcer index of the ulcerated group—Ulcer in the ex of treated group × 100)/Ulcer index of the ulcerated group [47]. 

### 4.7. Measurement of Oxidative Stress and Antioxidant Enzymes

After photographing, the stomach tissues were homogenized with ice-cold phosphate-buffered saline, and then centrifuged at 3000 rcf for 10 min. The supernatant was collected to evaluate SOD, GPx, GSH, MDA, and TAS–TOS levels. SOD activity was assessed as suggested by Sun et al. [48]. CAT activity was measured as specified by Aebi [49]. GPx activity was evaluated in line with Paglia and Valentine’s methods [50]. GSH activity was assessed as stated by Sedlak and Lindsay [51]. Protein levels were determined with the method of Lowry et al. [52]. Lipid peroxidation was evaluated by determining levels of malondialdehyde (MDA), as specified in the method of Placer et al. [53]. TAS–TOS analyses were conducted based on the manufacturer’s guidelines [54].

### 4.8. Measurement of Inflammatory Markers and Cytokines

Myeloperoxidase (MPO) activity was assessed following the method of Bradley [55]. Levels of IL-1 β, IL-6, and TNF-α cytokines were measured in homogenates of the gastric tissue using a rat ELISA kit, as stated in the manufacturer’s directions. Briefly, the samples were added to wells and incubated for 90 min at 37 °C. The liquid was removed, and biotinylated detection Ab working solutions were added and incubated for 60 min. Then, the plate was washed with horseradish peroxidase conjugate working solution and incubated for 30 min at 37 °C. Finally, substrate reagent and stop solution were added, respectively, and the plate was read at 570 nm using the Multiskan™ GO Microplate Spectrophotometer reader (Thermo Scientific, Waltham, MA USA).

### 4.9. Histopathology

Gastric tissues were fixed in 10% formalin solution for 48 h, then processed through routine follow-up procedures, including dehydration and removal of fixative and fixed-in paraffin blocks. Sections (dimensions: 5 μm) were stained with hematoxylin and eosin (H and E) and examined under a light microscope (Olympus BX51, Tokyo, Japan) for histological evaluations. Sections were evaluated as no (−), mild (+), moderate (++), and severe (+++), according to their histopathological features.

### 4.10. Immunostaining Analyses

Immunostaining analyses were performed as previously described in procedure 41. The stained sections were examined with a light microscope (ZEISS Axio, GER-MANY). IL-33 primary antibody was dripped into the slides at a ratio of 1/100 and incubated at 21 °C for 45 min. A semiquantitative scoring system was used, as follows: −, absent; +, very mild; ++, mild; +++, moderate; ++++, strong [56]. 

### 4.11. Double-Immunofluorescence Assays

Gastric tissue sections placed on adhesive slides were deparaffinized, dehydrated, and used for immunoperoxidase analysis. Endogenous peroxidase was inactivated with 3% H_2_O_2_. Then, the tissues were boiled in 1% antigen retrieval (citrate buffer; pH:6.1;100X) solution and allowed to cool at room temperature. Sections were incubated with protein block for 5 min to prevent non-specific background staining in tissues. The sections were incubated with primary antibody (Nrf-2) and kept in the dark for 45 min. Immunofluorescence secondary antibody (FITC) was used as a secondary marker and kept in the dark for 45 min. Then, the gastric tissues were incubated with the second primary antibody (HO-1). A secondary immunofluorescence antibody was employed as a secondary marker (Texas Red) and kept in the dark for 45 min. DAPI with the mounting medium was dripped onto the sections and kept in the dark for 5 min, and the sections were closed with a coverslip. The stained sections were analyzed with a fluorescent microscope (ZEISS Axio, Germany). 

### 4.12. Statistical Analysis

Data were analyzed with the SPSS 20.00 program (SPSS Inc., Chicago, IL, USA). Shapiro–Wilk and Levene tests were used to determine normality and homogeneity. The groups were compared using one-way analysis of variance (ANOVA) under parametric conditions, and the post-hoc comparison was made using the Tukey test. Immunohistochemical and immunofluorescence staining produced images, and 5 randomly chosen regions from each image were assessed using the ZEISS Zen Imaging Software (zen 2 core software, www.ziess.com, accessed on 1 July 2015) to determine the degree of positive staining. Data were statistically defined as mean and standard error (mean ± SE) for % area. Immunohistochemical findings were exposed to Kruskal–Wallis with Mann–Whitney U post-hoc test (*p* < 0.05). Data were shown as mean ± SD.

## 5. Conclusions

The present study highlights the role of the Nrf2/HO-1 signaling pathway/-IL 33 in IND-induced gastric ulcer formation. For the first time, we present scientific arguments suggesting the gastroprotective effects of SN berry extract. Indeed, the antiulcer activity of SN berries and their gastroprotective parameters were similar to those of the control drug, ESO. The suggested mechanisms of SN action are related to its ROS-scavenging properties that facilitate cell antioxidant properties and anti-inflammatory properties associated with the Nrf2/HO-1/IL-33 signaling pathways. This study has some limitations, including detailed analyses of HPLC components, and identifying the effects that discrete SN berry extract compounds incur in the in vivo gastric model. Further study is required to determine SN’s potential protective effects in other gastric ulcer models and medically as an alternative or adjuvant therapy for managing gastric ulcers. 

## Figures and Tables

**Table 1:** Results of the GC-MS chromatogram analysis.

	R.T. ^a^	Area ^b^	R.A. (%) ^c^	Compound Name	CAS ^d^	SI (%) ^e^
1	7.853	909,102	0.45	2,5-Dimethyltetrahydrofuran	1003-38-9	94
2	9.611	743,461	0.37	3-Hexanone	589-38-8	96
3	9.728	1,084,622	0.53	2-Hexanone	591-78-6	97
4	9.882	230,105	0.11	3-Hexanol	623-37-0	94
5	10.963	208,713	0.1	Isovaleric acid	503-74-2	95
6	14.770	9,605,684	4.72	Dimethipin	55290-64-7	96
7	15.089	11,505,459	5.66	Aldicarb	116-06-3	96
8	15.636	3,910,438	1.92	1-Butenyl methyl ketone	763-93-9	91
9	39.642	754,109	0.37	Ethyl myristate	124-06-1	94
10	39.807	950,844	0.47	n-Tetracosane	646-31-1	93
11	40.762	282,753	0.14	Neophytadiene	504-96-1	93
12	42.572	4,416,769	2.17	Methyl palmitate	112-39-0	95
13	43.263	9,498,091	4.67	L-(+)-Ascorbyl dipalmitate	28474-90-0	91
14	43.990	20,970,704	10.31	Ethyl palmitate	628-97-7	94
15	46.150	7,407,006	3.64	Methyl linoleate	112-63-0	94
16	46.669	874,377	0.43	Methyl stearate	112-61-8	94
17	47.446	34,862,172	17.14	Ethyl linoleate	544-35-4	92
18	47.594	24,967,197	12.27	Ethyl linolenate	1191-41-9	95
19	47.804	2,458,265	1.21	Octadecanamide	124-26-5	92
20	47.942	5,129,745	2.52	Ethyl stearate	111-61-5	91
21	49.900	276,963	0.14	n-Dotriacontane	544-85-4	91
22	51.210	2,094,842	1.03	Oleoamide	301-02-0	93
23	57.200	4,562,804	2.24	Tetrapentacosan	5856-66-6	92

^a^ Retention time, ^b^ Peak area, ^c^ Relative abundance, ^d^ Registry number, ^e^ Similarity Index.

**Table 2:** Quantitative analysis results the of SN berry extract sample.

No	Analytes	R.T. ^a^ (min)	Parent Ion ^b^	Fragment Ion ^c^ (m/z)	CE ^d^ (eV)	Polarity	Quantification (µg Analyte/G Extract)
1	Gallic acid	3.387	169.0 [M−H]^−^	125.1	12	Negative	32.82
2	Protocatechuic acid	5.411	153.0 [M−H]^−^	109.0	14	Negative	21.12
3	Chlorogenic acid	6.818	353.1 [M−H]^−^	191.0	12	Negative	1783.38
4	Caffeic acid	6.964	178.9 [M−H]^−^	135.1	14	Negative	89.30
5	Hydroxybenzaldehyde	7.412	121.0 [M−H]^−^	92.0	24	Negative	166.09
6	Epigallocatechin	7.769	307.0 [M+H]^+^	139.0	8	Positive	2.53
7	Catechin	7.852	288.9 [M−H]^−^	245.1	12	Negative	7.87
8	o-coumaric acid	7.945	163.0 [M−H]^−^	119.1	10	Negative	145.02
9	Taxifolin	8.191	304.8 [M]	258.9	12	Positive	746.71
10	Trans-ferulic acid	8.441	193.1 [M−H]^−^	133.9	12	Negative	217.21
11	Vanillic acid	8.758	167.0 [M−H]^−^	151.8	12	Negative	364.94
12	Salicylic acid	9.479	137.0 [M−H]^−^	93.1	18	Negative	211.07
13	Syringic acid	9.581	197.1 [M−H]^−^	181.8	10	Negative	383.32
14	Vanillin	9.687	153.0 [M+H]^+^	125.0	6	Positive	25.90
15	Sinapic acid	10.425	223.1 [M−H]^−^	208.0	10	Negative	45.12
16	Protocatechuic acid ethyl ester	11.324	181.0 [M−H]^−^	107.9	20	Negative	9.83
17	p-coumaric acid	11.513	163.0 [M−H]^−^	119.0	10	Negative	11.10
18	Quercetin-3-xyloside	12.363	432.7 [M−H]^−^	299.5	24	Negative	7.98
19	Kaempferol-3-glucoside	13.055	448.8 [M]	286.9	10	Positive	5.03
20	Fisetin	13.217	287.0 [M+H]^+^	212.9	32	Positive	16.77
21	Baicalin	13.653	446.8 [M]	270.9	16	Positive	6.01
22	Chrysin	14.169	254.9 [M+H]^+^	153.0	32	Positive	16.93
23	Quercetin	14.847	300.8 [M−H]^−^	179.0	20	Negative	55.18
24	Naringenin	14.986	270.9 [M−H]^−^	119.1	24	Negative	35.28
25	Biochanin A	15.718	284.9 [M+H]^+^	151.9	28	Positive	1.16
26	Hesperetin	16.599	300.9 [M−H]^−^	164.0	20	Negative	22.93
27	Kaempferol	17.173	284.9 [M−H]^−^	116.9	44	Negative	105.52
28	Diosgenin	23.382	415.0 [M+H]^+^	271.0	18	Positive	37.54

^a^ Retention time, ^b^ Molecular ion of the standard compound (mass-to-charge ratio), ^c^ Fragment ion of the standard compound (mass-to-charge ratio), ^d^ (CE) refers to collision energies of related fragment ion.

**Table 3:** Ulcer score, ulcer index, and effect of SN berries (preventive index).

	Ulcer Score	Ulcer Index	Preventive Index
Control	-	-	-
IND	25 ± 1.09 ^a^	2450	-
IND + ESO	1.42 ± 0.74 ^b^	145	%92.28
IND + SN	1.75 ± 0.93 ^b^	160	%90.14

Data shown for ulcer scores are mean ± SE (n = 7 rats/group, different letters in columns (a,b) show a statistical difference (*p* < 0.05)).

**Table 4:** Scoring of macroscopic and histopathological findings.

	Control	IND	IND + ESO	IND + SN
Bleeding in mucosa	-	+++	+	+
Erosion of mucosa epithelium	-	+++	+	+
Necrosis of mucosa epithelium	-	+++	+	+
Inflammation	-	+++	+	+
Hyperemia	-	+++	+	+
Edema in submucosa	-	+++	-	+

**Table 5:** Scoring of immunohistochemical findings.

	Control	IND	IND + ESO	IND + SN
**IL-33**	21.48 ± 1.18 ^a^	96.52 ± 3.16 ^b^	38.76 ± 2.28 ^c^	41.15 ± 2.12 ^c^

Data shown for ulcer scores are mean ± SE (n = 7 rats/group, different letters (a,b,c) in columns show statistical differences (*p* < 0.05)).

**Table 6:** Scoring of immunohistochemical findings.

	Control	IND	IND + ESO	IND + SN
Nrf-2	98.64 ± 4.26 ^b^	41.16 ± 1.17 ^a^	65.35 ± 3.78 ^c^	62.63 ± 2.06 ^c^
HO-1	92.66 ± 3.61 ^b^	35.24 ± 1.58 ^a^	58.74 ± 2.44 ^c^	55.12 ± 2.15 ^c^

Data for ulcer scores are expressed as means ± SE (n = 7 rats/group, different letters (a,b,c) in rows show statistical differences (*p* < 0.05)).

**Table 7:** Temperature program applied in the analysis of the SN berry extract.

Rate (°C/min)	Temperature (°C)	Hold (min)
-	50.0	2.0
10.0	300.0	5.0
3.0	320.0	1.0

## Data Availability

Requests for data will be considered by the corresponding author.

## Supplementary Materials

The supporting information can be downloaded at: https://www.mdpi.com/article/10.3390/ijms242015486/s1.
