# Supplementary material for: Flavonoid-Rich Sambucus nigra Berry Extract Enhances Nrf2/HO-1 Signaling Pathway Activation and Exerts Antiulcerative Effects In Vivo
Source: Int J Mol Sci. 2023 Oct 23;24(20):15486. doi: 10.3390/ijms242015486 (PMC10607857; doi:10.3390/ijms242015486)
Supplement: Supplementary file 1 [file ijms-24-15486-s001.zip › ijms-2600773-supplementary.pdf]

Supplementary file

GC-MS Analysis

Sample Preparation

50 mg of extract was taken into a tube and 1 mL of hexane was added. After 2 minutes of vortexing, the upper phase was filtered with a 0.45 µm PETF filter and transferred to a vial and analyzed in the GC-MS system.

GC-MS Analysis

The system used in the analysis is Shimatzu QP2010 Ultra Gas Chromatography-Mass Spectroscopy. 60 mx0.25 mmx0.25µm (Teknokroma-TBR 5MS) film column was used as column. Flow was 1.1 mL / min, injection volume was 1 µL and injection temperature was set at 250 °C. Electron Impact ionization was used as ionizator and the scanning range of the mass analyzer was set as 40-600 m/z. The temperature program applied in the analysis is as **Table 1.**

The chromatogram image (Figure 1) and the results (Table 2) of the analyzed sample are as follows (Compounds with 90% or more similarity in library matching are listed).

LC-MS/MS analysis

Sample Preparation

50 mg of sample extract was taken into a tube and 1 mL of water-methanol mixture (50:50) was added. The tube was sonicated for 5 min, and then the solution was filtered through a 0.25 µm PETF filter. The filtered solution was diluted 1:10 with a water-methanol (50:50) mixture and the solution was transferred to a vial for analysis.

Quantitative analysis of phenolic and flavonoid compounds by LC-MS/MS

The analysis of phenolic and flavonoid content was carried out with Agillent brand 6460 Triple Quad model liquid chromatography- tandem mass spectroscopy. C18 column (250 mm x 4.6 mm, 5µm) was used for chromatographic separation. The flow rate was 0.4 mL/min and the injection volume was 10 µL. The column temperature was set to 30°C. 0.1% (v/v) formic acid solution (A) and methanol (B) were used as the mobile phase. The gradient system used in the analysis is shown in Table S1. A standard chromatogram, Supplementary Fig. 1, was obtained with pure standards, and the amounts of analytes in the sample were determined by creating a calibration curve (Table 2).

Table S1. The gradient system used in the analysis.

	**Time**	**A**	**B**
1	3.00 min	75.0 %	25.0 %
2	12.00 min	50.0 %	50.0 %
3	16.00 min	10.0 %	90.0 %
4	21.00 min	10.0 %	90.0 %
5	24.00 min	97.5 %	25 %

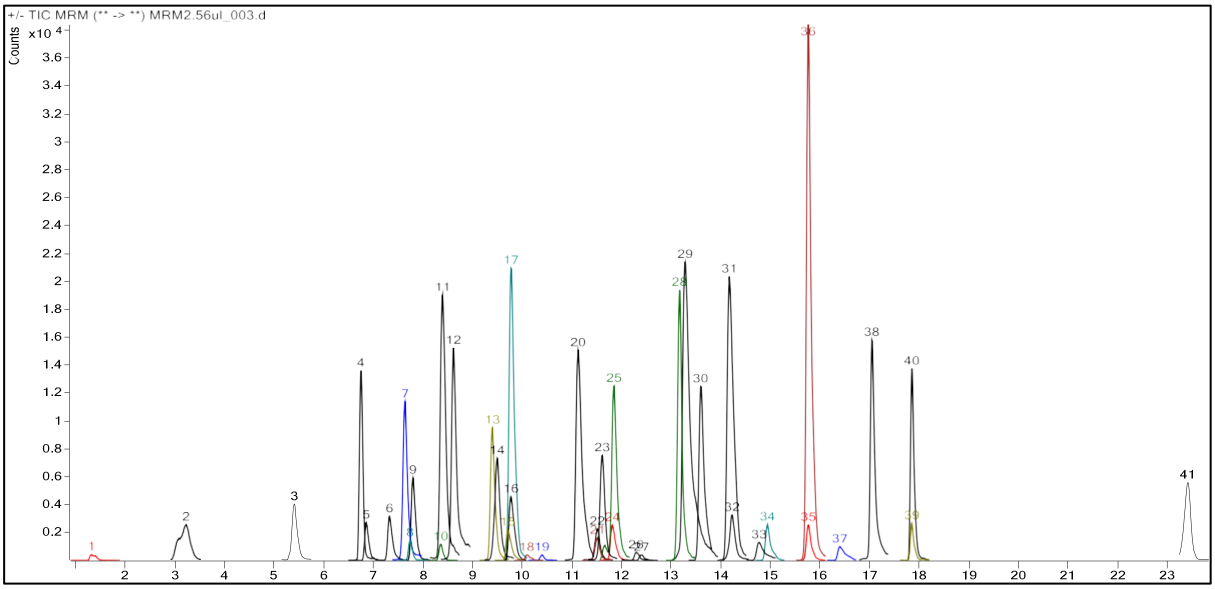

Supplementary Figure S1. MRM chromatogram of standard compounds : shikimic acid (1), gallic acid (2), protocatechuic acid (3), chlorogenic acid (4), caffeic acid (5), hydroxybenzaldehyde (6), epigallocatechin (7), catechin (8), o-coumaric acid (9), taxifolin (10), trans-ferulic acid (11), vanillic acid (12), salicylic acid (13), syringic acid (14), vanillin (15), caffein (16), polydatine (17), resveratrol (18), sinapic acid (19), scutellarin (20), protocatechuic ethyl ester (21), p-coumaric acid (22), coumarin (23), isoquercitrin (24), hesperidin (25), rutin (26), quarcetin-3-ksilozid (27), kaempferol-3-glucoside (28), fisetin (29), baicalin (30), chrysin (31), trans-cinnamic acid (32), quercetin (33), naringenin (34), biochanin a (35), morin (36), hesperetin (37), kaempferol (38), baicalein (39), luteolin (40), diosgenin (41).
